# Selective targeting of tumor associated macrophages in different tumor models

**DOI:** 10.1371/journal.pone.0193015

**Published:** 2018-02-15

**Authors:** Bianca Kakoschky, Thomas Pleli, Christian Schmithals, Stefan Zeuzem, Bernhard Brüne, Thomas J. Vogl, Horst-Werner Korf, Andreas Weigert, Albrecht Piiper

**Affiliations:** 1 Department of Medicine 1, University Hospital Frankfurt, Frankfurt, Germany; 2 Institute of Biochemistry I, Goethe-University Frankfurt, Frankfurt, Germany; 3 Department of Diagnostic and Interventional Radiology, University Hospital Frankfurt, Frankfurt, Germany; 4 Institute of Anatomy 2, University Hospital Frankfurt, Frankfurt, Germany; Universita degli Studi di Pavia, ITALY

## Abstract

Tumor progression largely depends on the presence of alternatively polarized (M2) tumor-associated macrophages (TAMs), whereas the classical M1-polarized macrophages can promote anti-tumorigenic immune responses. Thus, selective inhibition of M2-TAMs is a desirable anti-cancer approach in highly resistant tumor entities such as hepatocellular carcinoma (HCC) or breast cancer. We here examined whether a peptide that selectively binds to and is internalized by *in vitro*-differentiated murine M2 macrophages as compared to M1 macrophages, termed M2pep, could be used to selectively target TAMs in HCC and breast carcinoma. We confirmed selectivity of M2pep for *in vitro* M2 polarized macrophages. Upon incubation of suspended mixed 4T1 tumor cells with M2pep, high amounts of the TAMs were found to be associated with M2pep, whereas in mixed tumor cell suspensions from two HCC mouse models, M2pep showed only low-degree binding to TAMs. M2pep also showed low-degree targeting of liver macrophages. This indicates that the TAMs in different tumor entities show different targeting of M2pep and that M2pep is a very promising approach to develop selective M2-TAM-targeting in tumor entities containing M2-TAMs with significant amounts of the so far elusive M2pep receptor(s).

## Introduction

Mortality of patients with carcinomas such as breast carcinoma or hepatocellular carcinoma (HCC) is strongly determined by the aggressiveness of the tumor, which is, among others, determined by the tumor stromal compartment, containing i. e. fibroblasts, endothelial cells and infiltrating immune cells. In particular, tumor-associated macrophages (TAMs) are recognized to play a critical role in the regulation of the inflammatory microenvironment during carcinogenesis. The majority of TAMs resembles M2 or alternatively polarized macrophages that promote tumor progression by secreting pro-angiogenic and growth factors and by suppressing the adaptive immunity [1–3]. M2 macrophages are involved in tissue repair, mainly by providing growth factors [3]. In contrast, M1-polarized macrophages are pro-inflammatory and mediate anti-tumor responses. TAMs originate from circulating blood monocytes, which migrate towards the tumor environment and differentiate into M1 and M2 macrophages depending on the growth factors and chemokines [2]. The levels of M2-, but not M1-TAMs as well as of systemic CD163, which is shed from activated M2 macrophages, are indicators of unfavorable prognosis in cancer patients [4–8].

Due to their immunosuppressive and pro-tumorigenic properties, inhibition of TAMs is an anti-tumor strategy that is currently intensively explored. Non-selective depletion of macrophages e. g. by zoledronic acid in combination with sorafenib or inhibition of macrophage recruitment into HCCs inhibits HCC progression [9,10]. However, due to the important role of macrophages in normal physiology and pathophysiology, selective modulation of TAMs is desirable.

Different strategies have been used to eliminate TAMs or to repolarize them to the M1 state [11–15]. Nevertheless selective targeting of TAMs is a challenging task. Recently, a peptide has been identified, termed M2pep, which selectively binds to and internalizes into M2 as compared to M1 polarized mouse macrophages or other leukocytes [16]. Intravenously injected M2pep selectively targets TAMs in mice with CD26 colon carcinoma and 4T1 breast carcinoma xenografts [16,17], indicating that this approach is promising to enable selective targeting of M2-polarized TAMs once the receptor and its human homolog have been identified.

Here, we examined if M2pep specifically targets TAMs in mixed tumor cells from murine HCC and in breast carcinoma. We found that M2pep targets TAMs from the breast cancer mouse model with high potency. In two HCC mouse models M2pep also bound to the TAMs, but with much lower efficacy. Thus, M2pep is an important tool to develop selective TAM-targeting in tumor entities containing high amounts of M2pep binding sites.

## Materials and methods

### Peptide synthesis

M2pep and scM2pep were synthesized as previously described [16]. Both peptides were synthesized with a Lys_3_Gly_3_Ser linker and a C-terminal biotin tag and were purchased from GenScript USA Inc. The purity was more than 98%.

### Macrophage preparation and activation

Bone marrow-derived macrophages were obtained from male and female C57BL/6 wildtype mice (5–7 months old), which were received from the animal facility of the University Hospital Frankfurt. The mice were euthanized and both legs together with the hip were extracted. Tibias, femurs and the hip were individually flushed with sterile PBS, the cell suspension was collected, centrifuged and the pellet resuspended in ACK buffer (Lonza) to remove erythrocytes. The obtained white pellet was resuspended in DMEM containing 20 ng/ml macrophage colony-stimulating factor (M-CSF) (Biotrend) and 4×10^6^ cells per well were seeded on a 6 well plate. After 7 days of differentiation macrophages were activated overnight in DMEM containing 25 ng/ml interferon-γ (IFN-γ) (PeProtech) and 100 ng/ml lipopolysaccharide (LPS) (Enzo Life Science) for M1 cells; and 25 ng/ml interleukin-4 (PeProTech) for M2 cells. DMEM was supplemented with 10% fetal calf serum (Sigma-Aldrich) and 1% penicillin/streptomycin (Sigma-Aldrich).

### Immunofluorescence staining

5×10^5^ bone marrow-derived macrophages were cultivated on 8-well tissue culture chambers (Sarstedt). After 7 days of differentiation and overnight activation, the M1 and M2 macrophages were incubated with 200 μM M2pep or scM2pep for 30 min at 37°C. The peptides were labeled to Streptavidin-PE-CF594 (BD Horizon). In order to achieve higher signal intensity, we used 200 μM M2pep in the immunofluorescence experiments, which did not alter the ability of M2pep to discriminate between M1 and M2 macrophages. The immunostaining was done as previously described [18]. Anti-mouse F4/80 (1:50; eBioscience) and Phalloidin-Alexa 488 (Invitrogen) were used as primary antibodies. The secondary antibody for F4/80 was Alexa-Flour 633 (Invitrogen). Nuclei were stained with Hoechst 33342 (Sigma-Aldrich). All coverslips were mounted on slides with Permount toluene solution (Fisher Chemicals) and imaged using an Olympus Fluoview FV1000 confocal microscope.

### Gene expression analysis

For analyzing the expression of the M2 specific gene of arginase 1 (Arg1) and the M1 specific gene of the inducible nitric oxide synthase (iNOS), RNA of activated macrophages was extracted with the miRNeasy Mini Kit (Qiagen). Thereafter, the RNA was reverse transcribed using the cDNA synthesis kit for RT-qPCR (Thermo Scientific). Real-time PCR was done in duplicates using the QuantiTect SYBR Green PCR Kit (Qiagen). The PCR was carried out on a StepOnePlus instrument (Applied Biosystems) and the data was analyzed with the StepOne Software version 2.0. For the quantification of the Arg1 and iNOS expression levels the Tata-box binding protein (Tbp) was used as a house-keeping gene. The following primer sequences were used: mouse Arg1 -_*forward* 5‘-GTGAAGAACCCACGGTCTGT-3‘; *reverse—*5‘-CTGGTTGTCAGGGGAGTGTT-3‘; mouse iNOS -_*forward* 5‘-TGCATGGACCAGTATAAGGCAAG-3‘; reverse 5‘-CTCCTGCCCACTGAGTTCGTC-3‘; mouse Tbp -_*forward* 5‘-CTGACCACTGCACCGTTGCCA-3‘; reverse 5‘-GACTGCAGCAAATCGCTTGGGA-3‘.

### Binding of M2pep to isolated macrophages

First, the peptides M2pep and scM2pep were labeled with Streptavidin-Alexa 633. In parallel, the activated macrophages were harvested by Accutase (Sigma-Aldrich) treatment. Afterwards, both M1 and M2 macrophages were incubated with 20 μM of labeled peptide for 30 min at 4°C. Prior to flow cytometry analysis, the cells were stained with anti-mouse CD206-FITC (Biolegend) and anti-mouse CD86-PE (BD Bioscience) to determine the activation status of the macrophages (CD206 for M2 and CD86 for M1). Cells were analyzed on a BD LSR Fortessa (BD).

### Housing and animal care

All animal experiments were approved by the local animal care committee of the Regierungspräsidium Darmstadt and were in agreement with the German legal requirements (approval number FK/1062).

All animals used in the following procedures were housed in a clean, pathogen-free room in the animal facility of the University Hospital Frankfurt. All mice had free access to sterile food and water. The drinking water of the transforming growth factor-α (TGFα)/c-myc animals contained ZnCl_2_ in order to induce the hepatocarcinogenesis. In addition, all mice were allowed to acclimate for one week prior to beginning experiments.

For the injection of the tumor cells in the transplanted tumor models, the mice were anesthetized with isoflurane. For magnetic resonance imaging the mice were anesthetized by intraperitoneal injection of ketamine (70 mg/kg) and xylazine (12 mg/kg). For final experiments, the mice were anesthetized by intraperitoneal injection of ketamine (180 mg/kg) and xylazine (12 mg/kg). Thereafter, the anesthetized mice were killed by perfusion of the animals with HBSS, followed by the preparation of the cell suspensions from livers and tumors. For the preparation of the bone marrow-derived macrophages, the mice were euthanized by cervical dislocation.

### Generation of transplanted tumor models

HepG2 cells [19] and 4T1 cells were obtained from ATCC and were grown in DMEM supplemented with 10% fetal calf serum and 1% penicillin/streptomycin.

For generating HepG2 xenografted nude mice, 5×10^6^ cells were resuspended in 100 μl PBS and were injected subcutaneously into the flanks of 5 weeks old femal NMRI Foxn1 nude mice (Harlan Laboratories B.V.). The mice were observed twice per week. Once the tumors were visible by eye, the body weight of the animals were monitored and the tumor size was measured using calipers. Three to four weeks later, when the tumors reached a diameter of approximately 1 cm, the mice were sacrificed, the tumors excised and used immediately for the generation of single cell suspensions for following *ex vivo* experiments.

For generating 4T1 xenografts 8 weeks old female Balb/c mice (Harlan Laboratories B.V.) were used. 2.5×10^5^ cells in a volume of 10 μl of PBS were injected into the fat pad no. 4. For the injection, the fur was carefully incised and lifted to the site. Afterwards the fur was closed with clips. The mice were observed daily and body weight and tumor size were monitored. When the tumors reached a diameter of 0.75 cm, which normaly occured 6–7 days after the inoculation, the mice were sacrificed; the tumors were excised and used immediately for the generation of single cell suspensions for following *ex vivo* experiments.

During the experimental procedures no animal died ahead of schedule and no animal reached the target end points of a tumor diameter of >1.5 cm for HepG2 and >1 cm for 4T1 tumors, or a body weight loss of >20%. Furthermore, no adverse outcomes occurred such as blistering or ulceration of the tumors.

### Generation of TGFα/c-myc bi-transgenic mice with HCC

Male TGFα/c-myc bi-transgenic mice were generated by crossing homozygous metallothionein/TGFα and albumin/c-myc single transgenic mice in CD13B6CBA background [20–22]. Hepatocarcinogenesis was induced by ZnCl_2_ via the drinking water 4 weeks after birth. The animals were inspected once per week. Approximately 20–24 weeks after induction, HCCs were detected by contrast-enhanced magnetic resonance imaging as described recently [22,23]. Mice with visualized HCCs of a diameter of 0.5 to 0.8 cm were sacrificed, the tumors excised and used immediately for the generation of single cell suspensions.

Due to the monitoring of the endogenously formed HCCs by magnetc resonance imaging, HCCs were detected in the animals before they reached a diameter of >0.5 cm. All animals showed a normal fitness and activity, combined with no detectable weight loss. The tumor size was determined with the program Centricity RIS 4.1i Plus (General Electric Company, Frankfurt am Main, Germany) by measuring the length and the width of the detected tumors.

### *Ex vivo* binding study in suspended cells

For the *ex vivo* binding studies liver and tumor single cell suspensions were prepared simultaneously from the same animal. The tumor-bearing mice were perfused with collagenase via the vena cava as described previsously [24]. After removing the perfused liver from the abdominal cavity, it was placed in a petri dish on ice and opened with forceps. Liver cells were resuspended in DMEM. After two rounds of centrifugation (5 min at 50 × g and 4°C) and an additional centrifugation of supernatant for 7 min at 650 × g and 4°C the non-parenchymal liver suspension was obtained. In the TGFα/c-myc mice with HCC, HCCs remained undissociated after liver perfusion.

The undissociated HCCs from the TGFα/c-myc mice or the transplanted tumors were removed and used for the production of the tumor cell suspensions by the mouse tumor dissociation kit (Miltenyi Biotec). A total of 10^6^ cells of each suspension were incubated with 20 μM M2pep or scM2pep, earlier labeled to Streptavidin-Alexa 633, for 30 min at 4°C. For macrophage identification, to prevent unspecific antibody binding the cells were first treated with anti-mouse IgG (Jackson Immuno) and further stained with anti-mouse CD45-VioBlue (Miltenyi), anti-mouse Ly-6G-APC-Cy7 (Biolegend), anti-mouse F4/80-PE-Cy7 (Biolegend) and anti-mouse/human CD11b-eFlour 605 (Biolegend). Flow cytometry analysis was done on a BD LSR Fortessa (BD).

### Statistical analysis

Comparisons of the *in vitro* binding studies were performed with Wilcoxon-matched pair test. For *ex vivo* studies the Students t-test was used. In both cases P<0.05 was considered significant. Data were analyzed using the BiAS software for Windows (version 9.11, Epsilon-Verlag).

## Results

### M2pep selectively binds to and is internalized by *in vitro* differentiated murine M2 macrophages

**Fig 1:**
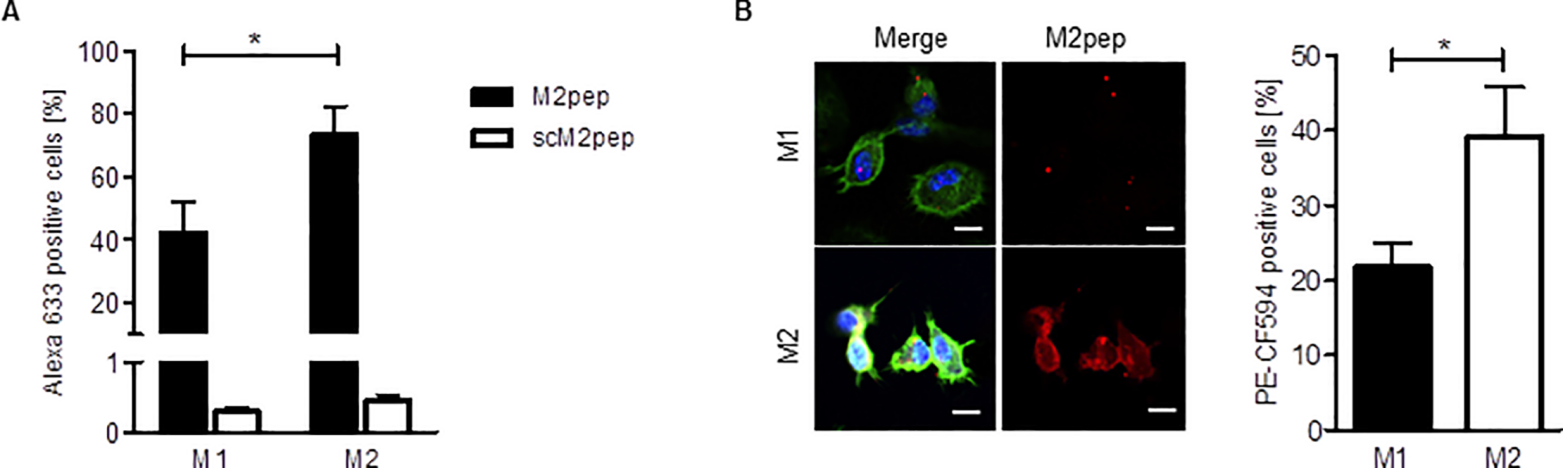
Specific binding of M2pep to and internalization into *in vitro* differentiated M2 macrophages. (**A**) M1 and M2 macrophages were incubated with M2pep or scM2pep (20 μM) for 30 min and analyzed by flow cytometry. (M2pep: n = 6; scM2pep: n = 4). (**B**) Representative immunofluorescence images of M1 and M2 macrophages incubated with 200 μM M2pep for 30 min (blue–Hoechst 33342; red–M2pep; green–phalloidin; white–F4/80; Scale 10 μm). The percentage of M2pep positive macrophages was quantified by counting the PE-CF594 positive cells in the M1 or M2 macrophage population (M1/M2 n = 10). Values are means ± SEM; *P<0.05. Statistical analysis was performed with the Wilcoxon-matched pair test.

As there was no independent validation of the finding that M2pep selectively binds to and internalizes into M2 macrophages, we polarized mouse bone marrow-derived macrophages with IFN-γ and LPS to M1 and with interleukin-4 to M2 macrophages. Immunophenotyping of the cells and fluorescence-activated cell sorting (FACS) analyses (CD206+ for M2 and CD86+ for M1) as well as analyses of the expression of arginase 1 (M2 marker) and iNOS (M1 marker) confirmed that these treatments led to the production of M1 and M2 macrophages, respectively (S1 Fig). Incubation of the cells with fluorescently labeled M2pep or a scrambled control peptide (scM2pep) and analysis of peptide binding to the cells by FACS analysis revealed much higher amounts of M2pep recognition by both M1 and M2 macrophages (Fig 1A). Approximately two-fold higher amounts of M2pep were associated with M2 macrophages as compared to M1 macrophages (Fig 1A). Imaging of the fluorescence of the peptide in the cells by confocal laser scanning microscopy shows that M2pep is also selectively internalized by M2 macrophages (Fig 1B), confirming that M2pep shows selective binding to and internalization into murine M2 macrophages.

### M2pep selectively targets TAMs in mixed HCC cell suspensions from TGFα/c-myc mice and HepG2 tumor xenografts

**Fig 2:**
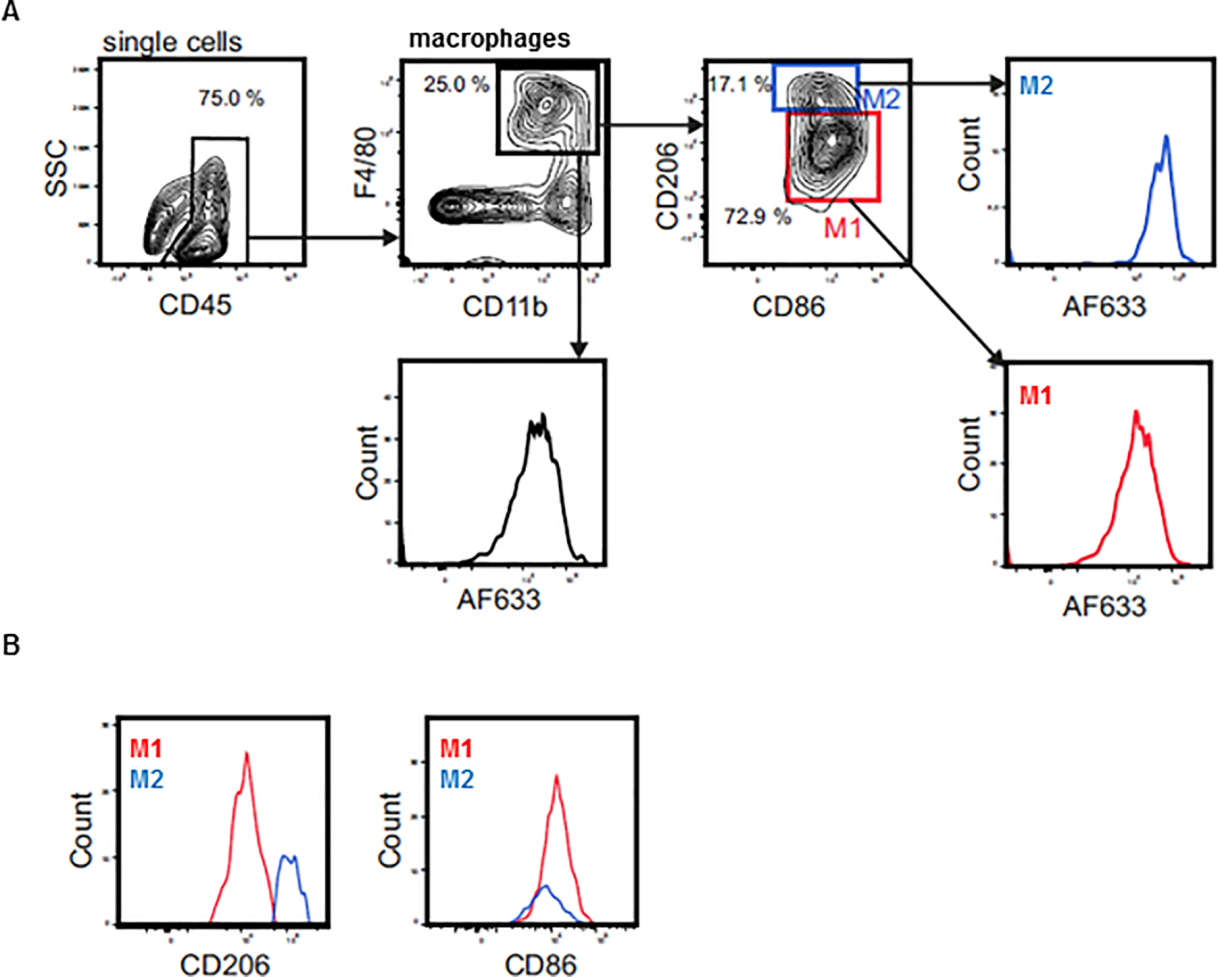
Gating strategy used in the FACS analyses to determine binding of M2pep to TAMs/liver macrophages in tumor and non-parenchymal liver cell suspensions. (**A**) Representative flow cytometry gating scheme. Liver macrophages or TAMs within extracted cell suspensions were identified by staining of CD45, F4/80 and CD11b. Discrimination between M1 and M2 marcophages was performed by their expression of CD206 and CD86, respectively. The Alexa 633 fluorescence intensity (AF633) was analyzed for all macrophages and for M1 an M2 macrophages in particular. Non-parenchymal liver cell as well as tumor cell suspensions were treated with 20 μM M2pep or scM2pep for 30 min on ice, followed by analysis for the Alexa 633 fluorescence intensity. (**B**) Representative histograms of the activation status of liver macrophages or TAMs within extracted cell suspensions. Shown are the flow cytometry results of the tumor cell suspension of the TGFα/c-myc mouse model.

**Fig 3:**
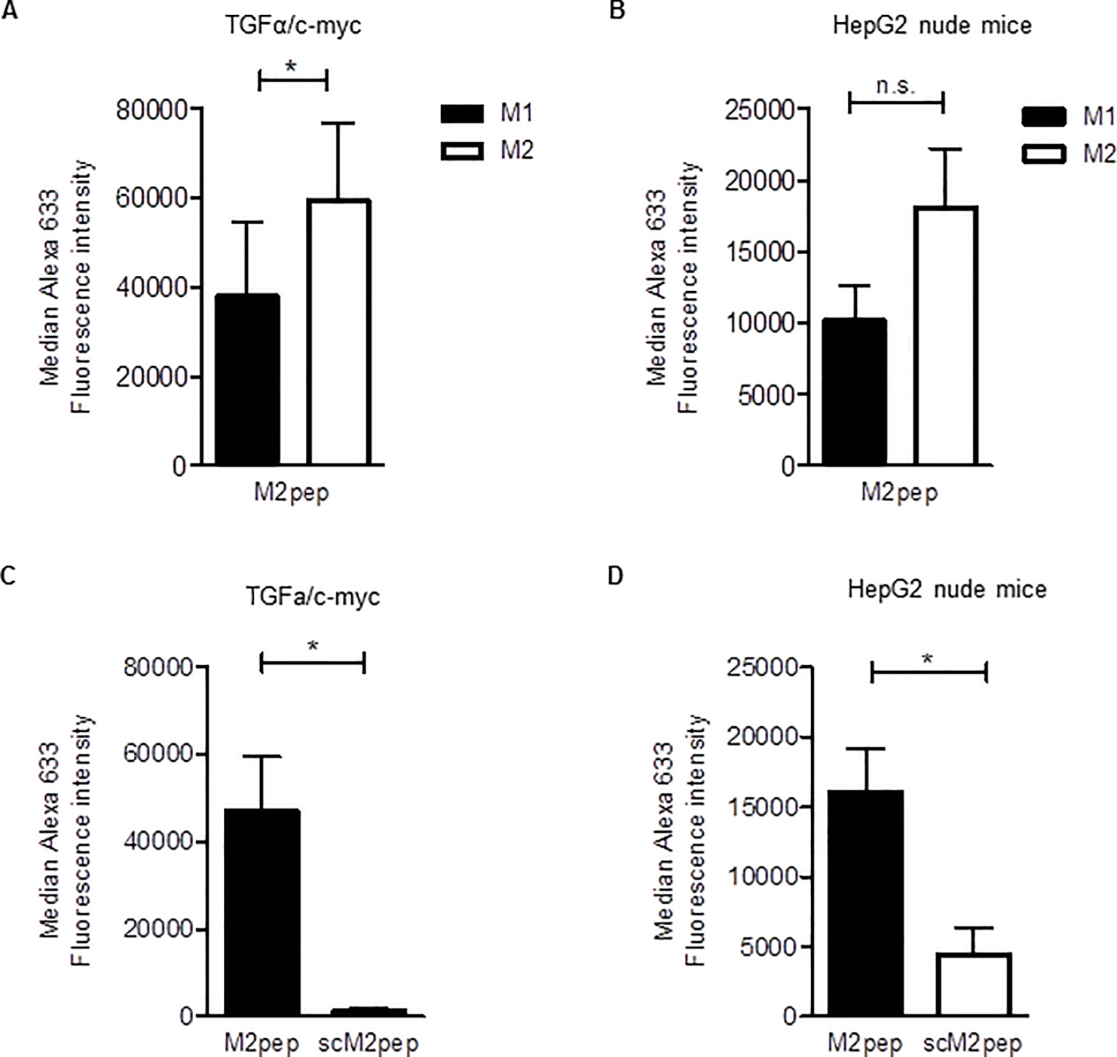
M2pep binding to TAMs in HCC cell suspensions. Tumor cell suspensions were treated with 20 μM M2pep or scM2pep for 30 min on ice, followed by analysis for the Alexa 633 fluorescence intensity. (**A**) and (**B**) Comparison of the median Alexa 633 fluorescence intensity of M2pep in M2 and M1 macrophages in tumor cell suspensions from (**A**) TGFα/c-myc mice (n = 4) and (**B**) HepG2 Xenograft nude mice (n = 5). (**C**) and (**D**) Comparison of the median Alexa 633 fluorescence intensity between tumor cell suspensions of (**C**) TGFα/c-myc mice (n = 4) and (**D**) HepG2 Xenograft nude mice (n = 5) treated with M2pep or scM2pep. Values are means ± SEM; *P<0.05. Statistical analysis was performed with Students t-test; n. s., not significant.

To investigate if M2pep shows selectivity for TAMs in HCC, we used resected HCCs formed endogenously in TGFα/c-myc transgenic mice as well as HepG2 xenografts excised from nude mice [21–23]. The resected tumors were dissociated and the suspended cells were incubated with fluorescently labeled M2pep for 30 min on ice. Thereafter, the amounts of fluorescence associated with TAMs were determined by FACS analysis (Fig 2A). Furthermore, the activation status of the macrophages within the cell suspension was verified by their expression of CD206 and CD86 (Fig 2B). As illustrated in Fig 3A and 3B, higher amounts of M2pep were found to be associated with M2-TAMs as compared to the M1-TAMs from HCC, although the selectivity of M2pep to associate with M2-TAMs as compared to M1-TAMs was modest, considering that TAMs in the HCC contained a higher portion of M2 macrophages as compared to M1 macrophages (S2 Fig). When the incubation was carried out with scM2pep instead of M2pep, only minimal amounts of the peptide were found to be associated with TAMs in both tumor models (Fig 3C and 3D).

### M2pep targets liver macrophages

**Fig 4:**
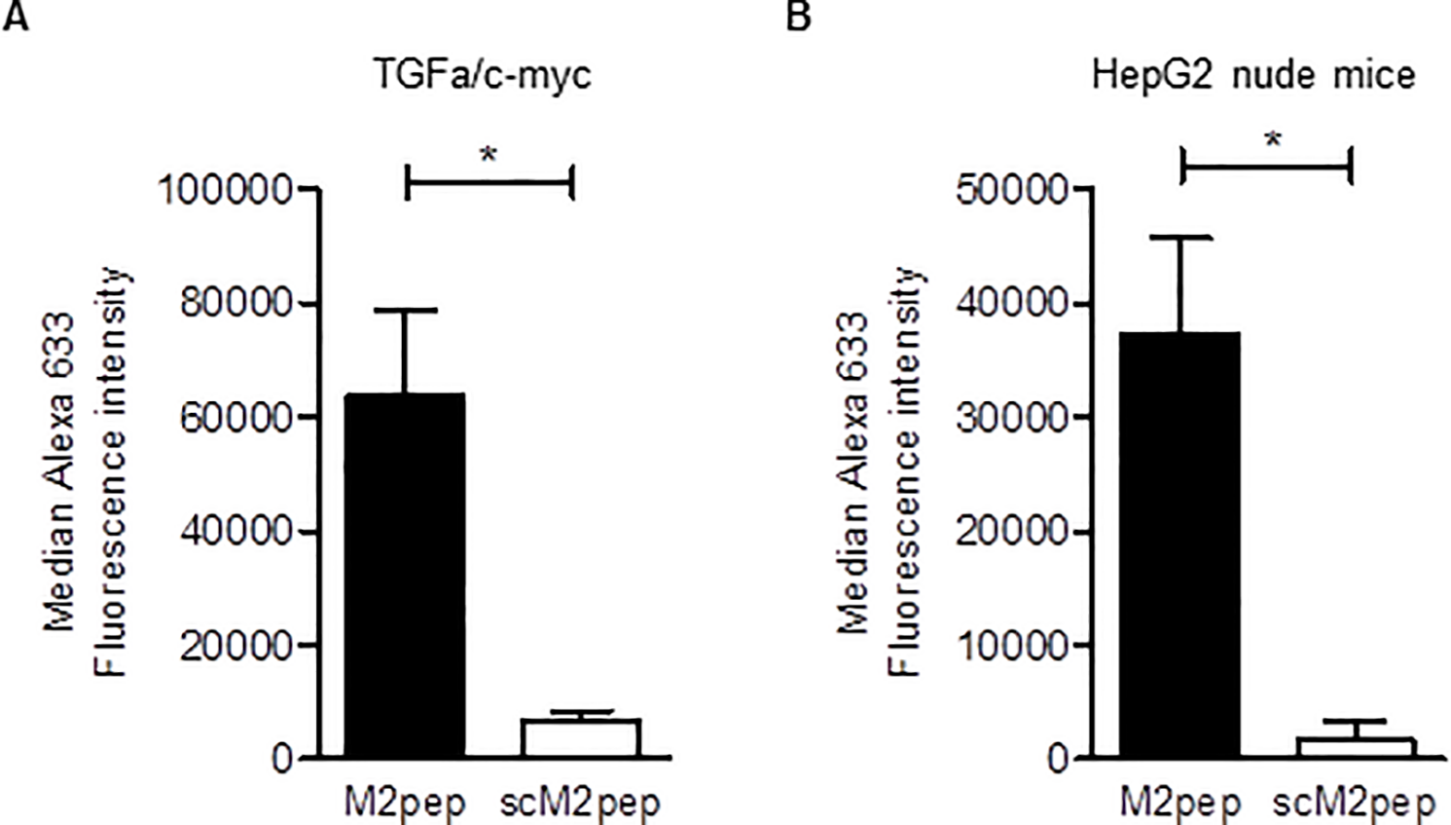
M2pep binding to macrophages in non-parenchymal liver cell suspensions. Suspended non-parenchymal liver cells obtained from (**A**) TGFα/c-myc mice (n = 4) and (**B**) HepG2 Xenograft nude mice (n = 5) were treated with 20 μM of Alexa 633-labeled M2pep or scM2pep for 30 min on ice. For the identification of the macrophages the cells were stained and analyzed by FACS analysis as described in Fig 2. Values are means ± SEM; *P<0.05. Statistical analysis was performed using Students t-test.

It is currently unclear whether M2pep also targets the resident macrophages in the liver (Kupffer cells), which show both M1 and M2 polarization [25]. Therefore, we investigated the association of M2pep with suspended non-parenchymal liver cells obtained from TGFα/c-myc mice as well as from HepG2 nude mice. As illustrated in Fig 4, M2pep showed considerable association with liver macrophages. When the incubation was carried out with scM2pep, much lower amounts of M2pep were associated with macrophages as compared to M2pep, indicating a specific binding of M2pep to liver macrophages.

### M2pep strongly targets TAMs in 4T1 tumors

**Fig 5:**
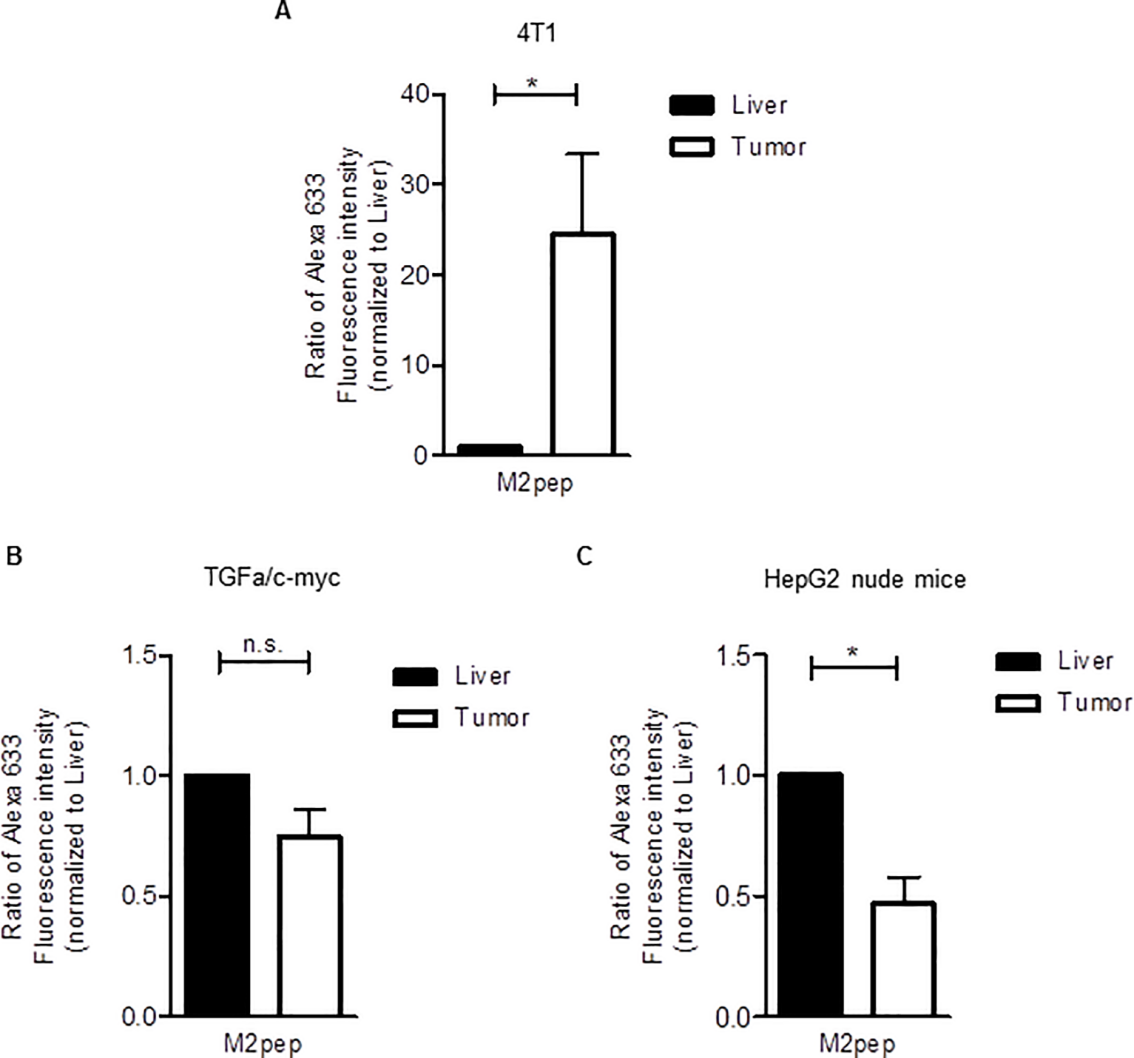
M2pep shows highly selective binding to TAMs in the dissociated 4T1 tumors that strongly exceeded that in the two dissociated HCCs. (**A**) Non-parenchymal liver cell and tumor cell suspensions of 4T1-bearing Balb/c mice were treated with 20 μM M2pep for 30 min on ice. Peptide binding was quantified based on the Alexa 633 fluorescence intensity by flow cytometry as described in Fig 2 (n = 3). (**B**) and (**C**) Non-parenchymal liver and tumor cells were prepared simultaneously from HCC-bearing (**B**) TGFα/c-myc mice (n = 4) or (**C**) HepG2 Xenograft nude mice (n = 5). The suspended cells were incubated with 20 μM M2pep for 30 min on ice. Subsequently, the cell suspensions were analyzed for association with M2pep by flow cytometry as described in Fig 2. Values are means ± SEM; *P<0.05. Statistical analysis was performed with Students t-test; n. s., not significant.

After we found modest binding of M2pep to the TAMs in HCC, we compared the ability of M2pep to target TAMs in HCCs with that in orthotopic 4T1 breast carcinoma tumors. To this end, mouse livers and tumors from the same animal were dissociated in parallel and the mixed tumor cells as well as the non-parenchymal liver cells were incubated with fluorescent M2pep. The amount of TAM-associated fluorescence was related to the amount of M2pep-labeled liver macrophages from the same animal. The latter served for internal normalization. Nevertheless, it should be noted that the numbers of labeled cells in the suspended non-parenchymal liver cells and tumor cells cannot be compared directly due to the differences in the preparation. As illustrated in Fig 5, in comparison to the M2pep labeling in the non-parenchymal liver cells, the labeling of TAMs by M2pep in the mixed 4T1 breast tumor cells by far exceeded that observed in the two HCC mouse models, even when taking in consideration the slightly higher amount of TAMs compared to liver macrophages in the 4T1 mouse model (S3 Fig), Thus, the ability of M2pep to specifically associate with TAMs in the suspended 4T1 tumor cells was much higher than that observed in the dissociated HCC cells.

## Discussion

Non-selective killing of macrophages has been shown to inhibit the progression of a number of tumor entities, including breast, colon, lung, ovarian and brain tumors [26], but this may have many undesired side-effects [3,27]. Moreover, as M1-TAMs are anti-tumorigenic, selective killing of M2-TAMs with preservation of the anti-tumorigenic M1-TAM function is likely to exert an additional anti-tumorigenic effect [27]. The present study indicates that M2pep is likely to enable the development of highly selective M2-TAM-targeting in tumor entities containing M2-TAMs with significant amounts of M2pep receptor(s).

After confirming that M2pep binds to and internalizes into M2 activated macrophages [16], we found that M2pep specifically and potently targeted TAMs in mixed tumor cells from dissociated 4T1 breast carcinomas as well as from two HCC mouse models, when compared to the ability of a scrambled peptide. The targeting efficacy of M2pep was much higher in the suspended breast cancer cells as compared to the suspended HCC cells. This suggests that the ability of M2pep to target TAMs varies strongly between different tumor entities and that M2pep might be more suitable to target TAMs in breast carcinoma as compared to HCC.

As M2pep shows selectivity for M2 macrophages and is internalized into the cells, it could be harnessed to specifically import pro-apoptotic peptides into the TAMs. Indeed, a fusion protein of M2pep with pro-apoptotic KLA sequences has been reported to kill TAMs in CD26 mouse colon carcinomas upon its intravenous injection [16]. However, we did not detect significant amounts of fluorescence in 4T1 tumors or HCCs upon injection of fluorescently labeled M2pep (0.6 μmol/kg of M2pep/kg) into mice with 4T1 tumors or HCC. As we could not inject higher doses of the peptide for technical reasons and M2pep has been reported to have a K_d_ of 100 μM to bind to M2 macrophages [16], it is possible that this failure was due to a too low concentration of M2pep in the blood in our *in vivo* experiments. The relatively low *in vivo* stability of M2pep [17] might be another reason for the failure to monitor M2pep in mice in our *in vivo* experiments.

Different from the study of Cieslewicz et al. [16], we observed specific binding of M2pep to Kupffer cells in the liver. Kupffer cells have a crucial role in both the pathogenesis and the resolution of various liver diseases and inflammatory states [28]. However, our data showing much higher M2pep binding to TAMs in suspended 4T1 tumor cells as compared to the liver suggest that it might be possible to largely avoid targeting of Kupffer cells, when the tumors contain TAMs with high amounts of M2pep binding receptors. This may help to avoid undesired targeting of the Kupffer cells, e. g. under conditions requiring resolving inflammation and regeneration in the liver. This might be an advantage in comparison to CSF-1R-directed approaches, which also target the liver macrophages [15]. On the other hand our data indicate that M2pep cannot be universally used to deplete M2-polarized TAMs in different tumor entities, as in our hands M2pep targeted TAMs and also showed considerable targeting of Kupffer cells in HCC.

Macrophages show considerable plasticity and thus differences with respect to their gene expression profiling, depending on their environment [2]. A likely explanation for the different targeting of M2 populations by M2pep is that they differ with respect to their M2pep receptor expression levels and that the different microenvironments in the liver and in different tumor entities account for the different M2pep receptor expression and, thus, binding and internalization.

The receptor for M2pep has not yet been identified. Its identification is likely to allow targeting of M2-TAMs by other approaches and strongly facilitate the further development of this promising approach of M2-TAM targeting. Moreover, this should lead to the identification of the human homolog of the M2pep receptor.

In summary, we found that a peptide that selectively targets M2-polarized murine macrophages shows significant differences in targeting TAMs in different tumor entities and it could be an important tool to develop selective TAM-targeting in tumor entities containing high amounts of M2pep binding sites. M2pep may therefore enable novel strategies to inhibit tumor progression and therapeutic resistance.

## Supporting information

S1 Fig Verification of macrophage activation. (**A**) Isolated murine bone-marrow macrophages were stimulated to turn into M1 or M2 macrophages overnight. The isolated RNA was then analyzed for the expression of iNOS or Arg1 by real-time PCR. Values are means ± SEM (M1: n = 6; M2: n = 7). (**B**) The activation status of isolated macrophages was quantified by measuring the fluorescence intensity of CD206-FITC and CD86-PE by flow cytometry. Dot plots are representative of three replicates. *P<0.05; ***P<0.001. Statistical analysis was performed using the Students t-test.
(TIF)

S2 Fig Characterization of macrophage polarization of the TAMs. Cell suspensions of tumors and livers from TGFα/c-myc mice (n = 4) (**A**) and HepG2 xenografted nude mice (n = 5) (**B**) were stained with CD45, F4/80 and CD11b, CD206 and CD86, followed by analyses by flow cytometry. Values are means ± SEM.
(TIF)

S3 Fig Characterization of macrophage populations. (**A**) Cell suspensions of tumors and livers from TGFα/c-myc mice (n = 4), (**B**) HepG2 xenografted nude mice (n = 5) and (**C**) 4T1 tumor-bearing mice (n = 3) were stained with CD45, F4/80 and CD11b to identify liver macrophages or TAMs and analyzed by flow cytometry. Values are means ± SEM.
(TIF)

## Abbreviations

FACS: fluorescence-activated cell sorting
HCC: hepatocellular carcinoma
IFN-γ: interferon-γ
iNOS: nitric oxide synthase
LPS: lipopolysaccharide
M-CSF: macrophage colony-stimulating factor
TAMs: tumor-associated macrophages
TGFα: transforming growth factor-α
