# Supplementary figures and images for: Selective targeting of tumor associated macrophages in different tumor models
Source: PLoS One. 2018 Feb 15;13(2):e0193015. doi: 10.1371/journal.pone.0193015 (PMC5814016; doi:10.1371/journal.pone.0193015)

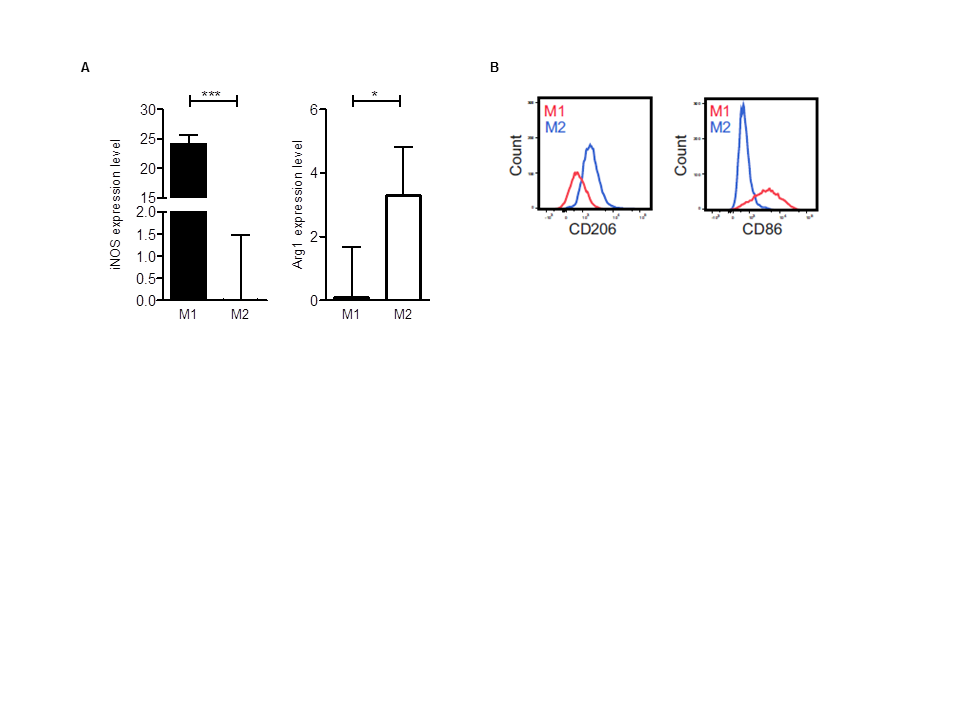

Supplement: S1 Fig — (A) Isolated murine bone-marrow macrophages were stimulated to turn into M1 or M2 macrophages overnight. The isolated RNA was then analyzed for the expression of iNOS or Arg1 by real-time PCR. Values are means ± SEM (M1: n = 6; M2: n = 7). (B) The activation status of isolated macrophages was quantified by measuring the fluorescence intensity of CD206-FITC and CD86-PE by flow cytometry. Dot plots are representative of three replicates. *P<0.05; ***P<0.001. Statistical analysis was performed using the Students t-test. (TIF) [file pone.0193015.s001.tif]

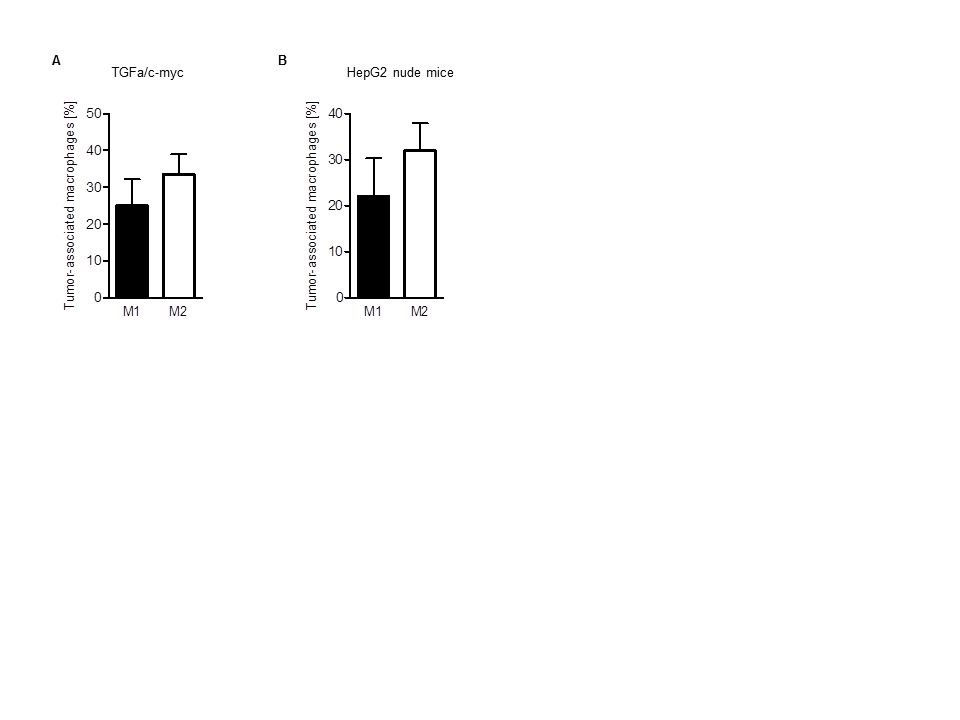

Supplement: S2 Fig — Cell suspensions of tumors and livers from TGFα/c-myc mice (n = 4) (A) and HepG2 xenografted nude mice (n = 5) (B) were stained with CD45, F4/80 and CD11b, CD206 and CD86, followed by analyses by flow cytometry. Values are means ± SEM. (TIF) [file pone.0193015.s002.tif]

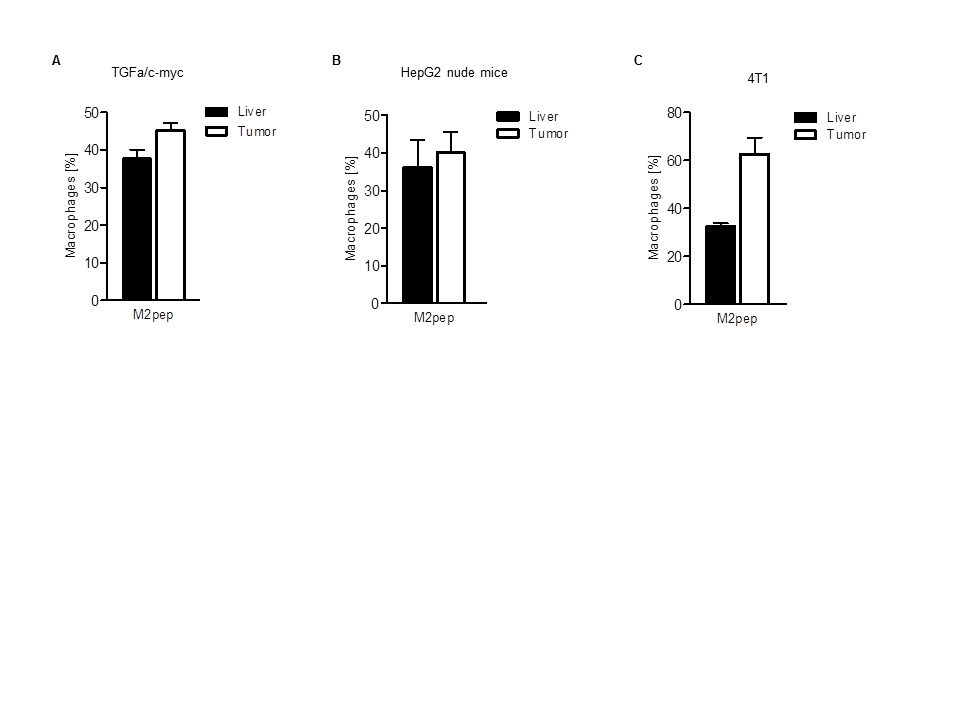

Supplement: S3 Fig — (A) Cell suspensions of tumors and livers from TGFα/c-myc mice (n = 4), (B) HepG2 xenografted nude mice (n = 5) and (C) 4T1 tumor-bearing mice (n = 3) were stained with CD45, F4/80 and CD11b to identify liver macrophages or TAMs and analyzed by flow cytometry. Values are means ± SEM. (TIF) [file pone.0193015.s003.tif]
